# Understanding Tick Biology and Its Implications in Anti-tick and Transmission Blocking Vaccines Against Tick-Borne Pathogens

**DOI:** 10.3389/fvets.2020.00319

**Published:** 2020-06-09

**Authors:** Biswajit Bhowmick, Qian Han

**Affiliations:** ^1^Key Laboratory of Tropical Biological Resources of Ministry of Education, Hainan University, Haikou, China; ^2^Laboratory of Tropical Veterinary Medicine and Vector Biology, School of Life and Pharmaceutical Sciences, Hainan University, Haikou, China

**Keywords:** anti-tick vaccines, *Ixodes*, *Borrelia*, transmission-blocking, blood, saliva

## Abstract

Ticks are obligate blood-feeding ectoparasites that transmit a wide variety of pathogens to animals and humans in many parts of the world. Currently, tick control methods primarily rely on the application of chemical acaricides, which results in the development of resistance among tick populations and environmental contamination. Therefore, an alternative tick control method, such as vaccines have been shown to be a feasible strategy that offers a sustainable, safe, effective, and environment-friendly solution. Nevertheless, novel control methods are hindered by a lack of understanding of tick biology, tick-pathogen-host interface, and identification of effective antigens in the development of vaccines. This review highlights the current knowledge and data on some of the tick-protective antigens that have been identified for the formulation of anti-tick vaccines along with the effects of these vaccines on the control of tick-borne diseases.

## Introduction

Ticks are found in the class of Arachnida, and they are classified into two families, hard ticks (*Ixodidae*) and soft ticks (*Argasidae*), each containing different genera and species of ticks (1, 2). Ticks are vectors of the causative agents of viral, bacterial and protozoan diseases in both animals and humans, and there has been an increasing incidence of several tick-borne pathogens (TBPs) in many parts of the world. After mosquitoes, ticks are considered to be the second most common vectors of pathogens to humans (3). They are competent vectors of disease-causing pathogens including *Borrelia burgdorferi sensu lato* (*s.l*.), *tick-borne encephalitis virus* (TBEV), and *Anaplasma phagocytophilum*. The most common tick-borne diseases and their vectors with different transmission cycles are presented in Table 1 (3–7). As ticks can transmit a variety of pathogens, an alternative control approach is to target the tick vector itself, either interfering with tick blood feeding or with pathogen transmission.

**Table 1:** Common tick-borne diseases and their vectors with different transmission cycles.

**Tick-borne pathogens (TBPs)**	**Main affected host**	**Tick-borne diseases (TBDs)**	**Time required for pathogen transmission**	**Main tick species**	**Transovarial transmission**	**References**
*Ehrlichia ruminantium*	Farm animal	Heartwater	48–96 h	*Amblyomma hebraeum, A. variegatum*	No	(3)
*Anaplasma platys*	Companion animal	Canine cyclic thrombocytopenia	16–72 h	*Rhipicephalus sanguineus*	No	(3, 4)
*Anaplasma phagocytophilum*	Human and farm animal	Human granulocytic anaplasmosis, tick-borne fever	24–48 h	*Ixodes ricinus*	No	(3, 4)
*Rickettsia rickettsii*	Human	Rocky Mountain spotted fever	10 h	*Dermacentor variabilis, D. andersoni, Rhipicephalus sanguineus, Amblyomma americanum*	Yes	(3–7)
*Anaplasma marginale*	Farm animal	Bovine anaplasmosis	24–48 h	*Rhipicephalus microplus, Dermacentor andersoni*	No	(3–7)
*Borrelia burgdorferi* (*sensu lato*)	Human and companion animal	Lyme disease	16–72 h	*Ixodes scapularis, I. pacificus*	No	(3–7)
*Crimean-Congo hemorrhagic fever virus*	Human and farm animal	Crimean-Congo hemorrhagic fever	Immediate	*Hyalomma impeltatum*	Yes	(3–7)
*Louping ill virus*	Farm animal	Louping ill	Immediate	*Ixodes ricinus*	No	(3–7)
*Tick-borne encephalitis virus*	Human	Tick-borne encephalitis	Immediate	*Ixodes ricinus, I. ovatus, I persulcatus*	Yes	(6)
*Theileria annulata*	Farm animal	Tropical theileriosis	48 h	*Hyalomma anatolicum anatolicum, Hyalomma anatolicum excavatum, Rhipicephalus appendiculatus*	No	(3)
*Babesia divergens, B. microti*	Human	Human babesiosis	48–72 h	*Ixodes persulcatus*	Yes/ No	(6)
*Babesia canis, B. vogeli*	Companion animal	Canine babesiosis	48 h	*Rhipicephalus sanguineus sensu lato* (*s.l*.), *Dermacentor reticulatus, Haemaphysalis leachi*	Yes	(3–7)
*Babesia divergens, B. jakimovi, B. bovis, B. bigemina, B. ovata*	Farm animal	Bovine babesiosis	48–216 h	*Rhipicephalus microplus, R. annulatus, Ixodes ricinus*	No	(3–7)

Globally, 1.3 billion cattle populations in the world are at risk of TBPs with an annual economic cost estimated to be US$22–30 billion in 1996 (8). This economic cost is going to be higher now in 2020 as it has been over 20 years. Currently, tick control depends mainly on the application of conventional chemicals or synthetic acaricides, resulting in acaricidal resistant tick populations in several developing countries and contamination of the environment and food products (9). Therefore, new approaches such as immunological control via vaccination are urgently required for effective control. Of which, vaccines seem to be a promising, safe, sustainable, cost-effective and environment-friendly solution. Only two ectoparasite vaccines were reported commercially in the early 1990s: The Australian TickGARD® (Hoechst Animal Health) and Latin American Gavac® (Heber Biotec). Both vaccines are derived from *Rhipicephalus microplus* midgut membrane-bound recombinant protein BM86 (10, 11). Despite the effectiveness of these commercial BM86-based vaccines for the control of cattle tick infestations, they show strain to strain variation in vaccine efficacy and are effective against *Rhipicephalus* tick species mainly (12). It has been nearly 30 years since the first anti-tick vaccines became commercially available. The identification of effective antigens remains to be the biggest hurdle in the development of further anti-tick vaccines. Thus, strategies to identify anti-tick vaccine antigen(s) should be based on expanding our knowledge of the biology of the ticks and its interaction with pathogens. This review will, therefore, focus on how the identification and functional characterization of selected tick proteins, with a particular focus on saliva, blood digestive and membrane-associated proteins, could help in the fight against tick-transmitted diseases and discuss the proteins suitability as anti-tick vaccine candidates.

## Saliva-assisted Transmission Blocking Vaccine Candidates

Saliva-assisted transmission (SAT) is defined as “the indirect promotion of arthropod-borne pathogen transmission via the actions of arthropod saliva molecules on the mammalian host” (13). This characteristic has been reported for most hematophagous arthropods including ticks. In this section, we review work that focuses on tick saliva proteins (TSPs) which are critical to the success of ticks as vectors of TBPs, and thus might serve as targets in tick antigen-based vaccines to prevent TBP infections. Saliva-assisted transmission blocking anti-tick vaccine candidates are listed in Table 2 (14–21).

**Table 2:** Saliva-assisted transmission blocking anti-tick vaccine candidates.

**Salivary molecule**	**Pathogen**	**Tick species**	**Target mechanism**	**Activity**	**References**
TSLPI	*Borrelia burgdorferi*	*Ixodes scapularis, I. ricinus*	Tick salivary lectin pathway inhibitor	Facilitates transmission from ticks to mice and from mice to ticks	(14)
tHRF	*B. burgdorferi*	*I. scapularis*	Tick histamine release factor (vasodilation)	Promotes late stage feeding and thereby facilitates tick to host transmission	(15)
Salp15	*B. burgdorferi*	*I. scapularis*	Secreted salivary protein (acquired immune responses)	Facilitates transmission from ticks to mice	(16)
Salp25 D	*B. burgdorferi*	*I. scapularis*	Salivary protein, antioxidant (acquired immune responses)	Facilitates transmission from mice to ticks	(17)
Salp16	*Anaplasma phagocytophilum*	*I. scapularis*	Secreted salivary protein (acquired immune responses)	Facilitates transmission from ticks to mice	(18)
Subolesin (SUB)	*B. burgdorferi*	*I. scapularis*	A reduction in tick weight and/or oviposition	Tick protective antigen (innate immune response, reproduction, and development of ticks)	(19, 20)
Subolesin (SUB)	*Tick-borne encephalitis virus*	*I. scapularis*	Nuclear factor kappa B (NF-kB)	Tick protective antigen (innate immune response, reproduction, and development of ticks)	(19, 20)
64P	*Tick-borne encephalitis virus*	*I. scapularis*	Dual-action (exposed and concealed)	Tick cement protein, secreted glycine-rich proteins	(21)
Sialo L2	*B. burgdorferi*	*I. scapularis*	Secreted salivary protein (acquired immune responses)	Increases level of skin infection following syringe inoculation	(54, 55)
Sialo L2	*phagocytophilum*	*I. scapularis*	Secreted salivary protein (acquired immune responses)	Protection by inhibiting inflammasome formation in mice	(54, 55)
Salp20	*Borrelia garinii*	*I. scapularis*	Secreted salivary protein (acquired immune responses)	Protection by inhibiting the complement pathway	(27)

Blocking attachment of a tick to the host is not only impairing blood feeding but also the transmission of tick-borne diseases (TBDs). The process of attachment starts with the intrusion of the tick's mouthparts into the host skin, followed by anchoring of the tick by a cement cone that mostly comprises secreted glycine-rich proteins. The molecular weight of 15-kDa tick cement protein, 64P, was first identified from the African brown ear tick *Rhipicephalus appendiculatus* that has been evaluated for its direct effect on TBEV transmission. Vaccination of mice with recombinant forms of 64P (64TRPs, expressed as truncated) significantly diminished TBEV transmission. In guinea pig, rabbit, mice and hamster models, these cement proteins act as a dual-action vaccine by targeting both “exposed” and “concealed” antigens, resulting in mortality of engorged ticks. Further, 64TPRs have been shown as a broad-spectrum vaccine candidate against adult and immature stages of several tick species, including *Ixodes ricinus* and *Rhipicephalus sanguineus* (22). The protective effect of anti-tick immunity against TBEV infection underpins the concept of transmission-blocking vaccines. Unlike 64TRP antigen, immunization with TickGARD did not provide protection against lethal infection with TBEV. Thus, 64TRPs antigens have the potential candidate for broad-spectrum transmission-blocking vaccines (21). Apart from 64TRPs, subolesin (SUB) is the second anti-tick vaccine candidate in the context of TBEV infection. It is an ortholog of vertebrate and tick akirins (AKR) that could be potentially considered as a universal vaccine against multiple blood-sucking arthropods including mosquitoes and ticks. Vaccination with recombinant subolesin protein showed a reduction of tick infestations and transmission of *B. burgdorferi, Anaplasma marginale, A. phagocytophilum*, and *Babesia bigemina* (19, 20). Havlíková et al. (23) found that SUB vaccines did not have any effect on TBEV infection and transmission. However, TBEV infection increases SUB mRNA transcript levels in tick tissues, thus supporting a role for this molecule in tick innate response to virus infection. Pilot studies have used *Babesia* and *Anaplasma* infection models to characterize SUB, TROSPA (identified in *Ixodes scapularis* as a receptor for *B. burgdorferi*) and SILK (a protein present in tick saliva) as potential antigens to control pathogen transmission in cattle. Rabbit polyclonal antibodies generated against SILK, TROSPA and SUB were added to infected or uninfected bovine blood to capillary feed *R. microplus* ticks. The results from capillary feeding showed a substantial effect on tick body weight, mortality, and oviposition rate, but infection levels were not changed in ticks treated with these antibodies (24). Because of the variations in infection levels from tick to tick, this experiment reported that artificial capillary blood feeding was not an ideal approach to characterize the efficacy of tick-pathogen interactions (24).

Salp15, a glycosylated salivary protein, was first identified from the black-legged tick *I. scapularis*. It is coded by 408 bases, and has a molecular weight of 14.7 kDa. The sequence analysis revealed weak similarities with two motifs of Inhibin A, a member of the TGF-ß super-family, and contained a signal peptide at N-terminal, suggesting that the protein may have immunosuppressive and secretory activity (21). It inhibits CD4^+^ coreceptor of mammalian T cells activation, dendritic cell-induced T cell activation through interaction with the C-type lectin and complement activity (25). Furthermore, the Salp15 protein binds to the outer membrane protein (OspC) of *B. burgdorferi* and protects the evasion of immune responses against spirochetes upon entry into the vertebrate host (16), facilitating long-lasting survival of the spirochetes, host infection and pathogen transmission. RNA interference-mediated (RNAi) silencing of Salp15 in infected *I. scapularis* significantly decreased the bacterial load in mice. RNAi studies provide the first direct evidence that Salp15 promotes TBPs transmission (16). Vaccination of mice with Salp15 showed substantial protection (60%) from *B. burgdorferi* infection (26), which provided further evidence. Following the discovery of Salp15-mediated transmission of *Borrelia* by *I. scapularis*, other salp15 homologs have been identified in *Ixodes persulcatus, Ixodes ricinus, Ixodes affinis*, and *Ixodes sinensis* ticks, they also bind *Borrelia garinii* and *Borrelia afzelii* OspC to facilitate spirochete transmission (26). Generally, high sequence similarity across various *Ixodes* species not only suggests an important role in blood feeding and tick–pathogen interactions but may also lead to the development of an anti-tick vaccine against TBDs.

Most salivary proteins are responsible for pathogen transmission from an infected tick to a host, but some saliva proteins are considered to aid in the acquisition of the pathogen by uninfected feeding ticks. One such protein is Salp25D, a 25-kDa tick salivary antioxidant protein expressed in both the salivary glands and midguts of *I. scapularis*. It has significant homology to glutathione peroxidase antioxidant that has been proven to play a critical role in protecting *Borrelia* from reactive oxygen produced by neutrophils and facilitating *Borrelia* acquisition by ticks (21). Knockdown of Salp25D significantly reduced acquisition of spirochetes by ticks engorging on *B. burgdorferi*-infected mice, but did not affect *Borrelia* transmission from infected nymphs to uninfected mice. Vaccination of mice with Salp25D also decreased the acquisition of *Borrelia* by ticks to three-fold in comparison to the control group (17). Hence, it seems that Salp25D facilitates the transmission of *Borrelia* from infected mice to uninfected ticks by protecting spirochetes from the toxic products of neutrophils activated by tick feeding (27).

Several other salivary proteins have been identified that play an important role in *A. phagocytophilum*-vector interactions. They include Salp16, alpha1-3-fucosyltransferase, P11 and an antifreeze glycoprotein (IAFGP) (21). Salp16, a 16-kDa protein, was identified in the salivary glands of *I. scapularis* and plays a role in *A. phagocytophilum*-vector interactions. Silencing of Salp16 induced significant (90%) reduction of *A. phagocytophilum* migration from gut to salivary glands through the haemolymph (18). Another study showed a significant increase in nuclear G-actin and phosphorylated actin upon *A. phagocytophilum* infection that subsequently influenced Salp16 gene expression (28). Two proteins, namely alpha 1-3-fucosyltransferase and IAFGP (*I. scapularis* tick antifreeze glycoprotein) were also found to be up-regulated upon the presence of *A. phagocytophilum* in ticks. RNAi-mediated knockdown of IAFGP and alpha 1-3-fucosyltransferase showed a significant reduction of *A. phagocytophilum* load in ticks when fed on infected mice (21). These results suggest the important role of these genes in tick-host interactions. P11, a secreted salivary protein, was up-regulated in *A. phagocytophilum*-infected ticks, which has been demonstrated in RNAi. The protein was shown to bind to *A. phagocytophilum* and facilitate the uptake of the pathogen by tick haemocytes, suggesting that haemocytes transport the pathogen from the midgut to the salivary glands (29).

Ticks have adopted a unique strategy to control the effects of host-derived histamine, and they secrete salivary molecules that neutralize the inflammatory effect of histamine (30). The tick histamine release factor (tHRF) was first characterized from *I. scapularis* using 2D fluorescence differential gel electrophoresis (DIGE). Real-time PCR analysis revealed that tHRF was expressed in both tick saliva and midgut and up-regulated in *Borrelia*-infected ticks. tHRF-silenced ticks or immunization of mice with recombinant tHRF significantly impaired the efficiency of tick feeding and spirochete transmission (15). As tHRF is critical for *I. scapularis* feeding irrespective of *B. burgdorferi* infection, and preferentially expressed between 48 and 72 h post-tick attachment (while *B. burgdorferi* s.s. transmission begins 36–48 h post-tick attachment), it appears that the effect on *Borrelia* transmission is due to the ability of tHRF to promote engorgement rather than to a specific interaction as with Salp15. Besides, increased vascular permeability effect of histamine may lead to the dissemination of *Borrelia* injected by the tick into the feeding site (15). Blocking tHRF might offer a viable strategy to develop vaccines that block tick feeding and therefore transmission of tick-borne pathogens. An *I. ricinus* ortholog of *I. scapularis* tHRF has been identified, but it had no effect on tick feeding upon vaccination. These results suggested that ticks have a multifaceted control mechanism for histamine and redundancy in the expression of histamine releasing factor proteins (31).

It has been shown that inhibition of the complement cascade at the tick bite site would not only be beneficial for the tick attachment but could also play a role in the survival and transmission of vector-borne pathogens in the mammalian host. Characterization of Salp15-assisted transmission of Lyme spirochetes demonstrates the importance of alternative and classical complement pathways in vertebrate host control of the spirochete. Unlike Salp 15, tick mannose-binding lectin inhibitor (TSLPI) does not bind to *B. burgdorferi* but instead interacts with the lectin-complement system, inhibiting complement cascade activation and reducing complement-mediated lysis of *Borrelia*. It is a glycosylated secretory salivary protein of 8 kD that was first identified from the salivary glands of *I. scapularis*. Expression of TSLPI mRNA levels is up-regulated in salivary glands 24 h after tick attachment compared to uninfected ticks, facilitating the survival of the spirochete and pathogen transmission. TSLPI-silenced ticks, or immunization of mice with anti-TSLPI antibodies, hampered *B. burgdorferi* transmission and acquisition by ticks (14). An ortholog from *I. ricinus* inhibits the lectin complement pathway and protects *B. garinii* and *B. burgdorferi* s.s. from complement-mediated killing *in vitro*, but TSLPI-assisted transmission of *B. burgdorferi* s.s. has not been identified. The saliva of *Ornithodoros* species inhibits both the alternative and classical pathways, while several *Ixodes* species (*Ixodes uriae* and *Ixodes hexagonus*) inhibit the alternative complement pathway, indicating that complement inhibitor in tick saliva is one of the important steps following tick attachment (13).

A recent study found that *Amblyomma americanum* ticks might utilize countervailing functions of pro- and anti-inflammatory proteins to regulate the evasion of host defenses. Both *in vitro* and *in vivo* data analyses demonstrated that the ticks first secreted pro-inflammatory proteins (insulin-like growth factor binding-related proteins: *Aam*IGFBP-rP6L, *Aam*IGFBP-rP6S, and *Aam*IGFBP-rP-1) that activated macrophage into the pro-host defense phenotype and then secreted anti-inflammatory proteins (serpins, AAS41, and AAS27) to deactivate the activated immune cells including macrophages (32). Thus, the discovery of tick saliva immuno-modulatory proteins is highly sought after as these might serve as targets in tick-antigen based vaccines to prevent TBPs.

## From Tick Biology To Anti-tick Vaccines

**Figure 1:**
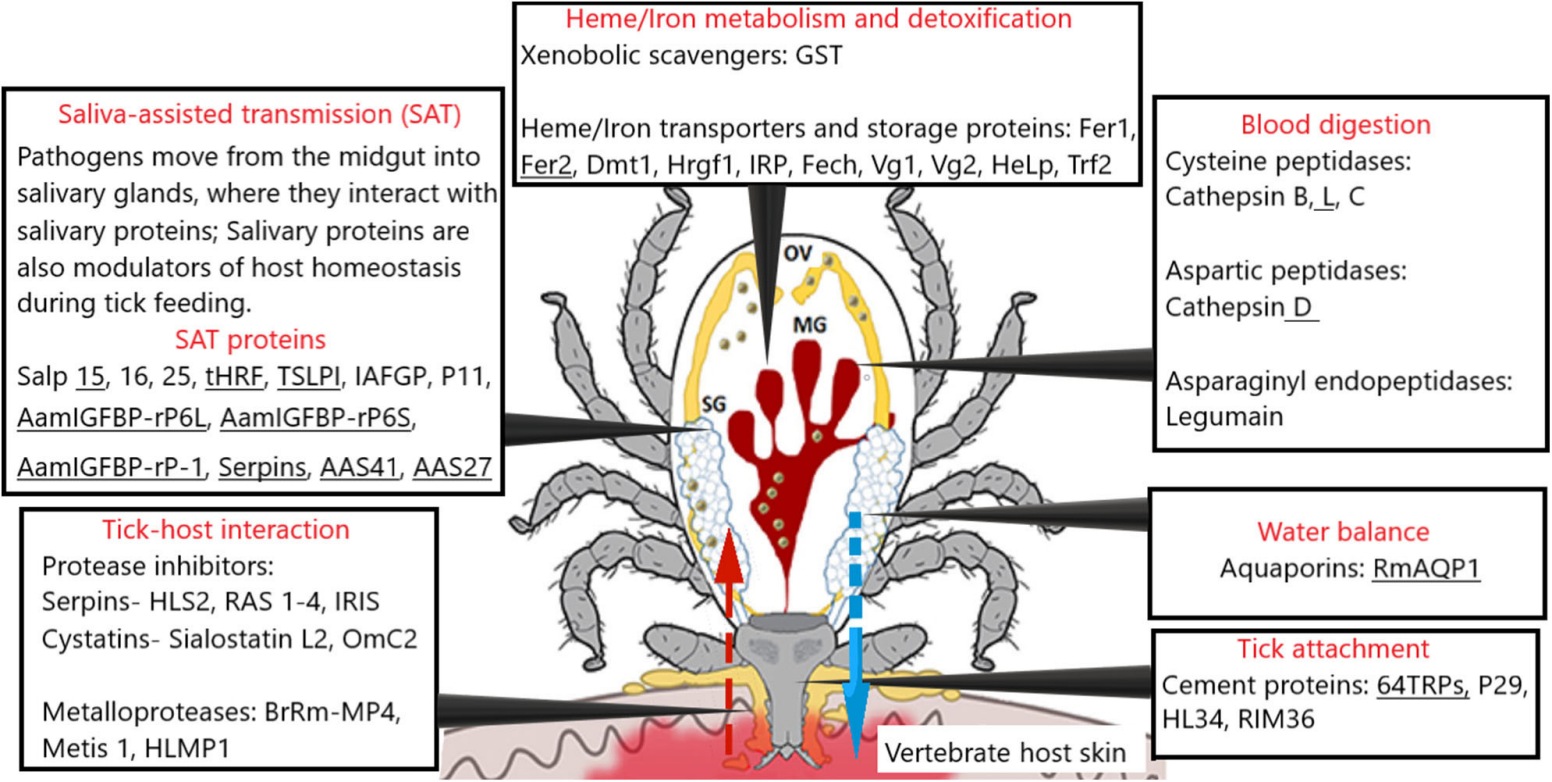
A schematic representation of tick physiological processes and involved molecules tested as vaccine candidates [modified from (78, 103)]. The most promising vaccine candidates are underlined. The red arrow represents blood meal uptake, and the blue arrow represents saliva injection. Several tick protease inhibitor families have been reported in the salivary glands and implicated in both tick biology/physiology. Major blood digestive enzymes (cathepsin B, C, D, L, and Legumain), blood digestion, heme/iron metabolism, detoxification, and inter-tissue transport that may serve as rational targets for “anti-tick” intervention. SG, salivary glands; MG, midgut; OV, ovary; HLS2, *Haemaphysalis longicornis* serpin-2; RAS-1,2,3,4, *Rhipicephalus appendiculatus*; IRIS, *Ixodes ricinus* immunosuppressor; Sialostatin L, *Ixodes scapularis*; OmC2, *Ornithodoros moubata*; Metis-1, metalloproteases from *Ixodes ricinus*; BrRm-MP4, metalloproteases *Rhipicephalus* (*Boophilus*) *microplus*; HLMP1, *Haemaphysalis longicornis* metalloprotease.

The tick biological processes are associated with host attachment, followed by feeding on the host, pathogen transmission to the host, intracellular blood digestion, nutrient extraction from the meal, as well as oviposition and egg-laying. Up to date, these approaches have offered several potential vulnerable targets for developing anti-tick vaccines (Figure 1). Successful vaccination against the tick has been previously achieved by targeting tick gut antigen, such as Bm86. In this section, we review work that has focused on saliva, transmembrane, and blood digestive proteins that are targeted to date and link the research findings to our current understanding and approaches for the development of anti-tick vaccines.

### Tick Salivary Protein Families With Anti-tick Vaccine Potential

Our knowledge of tick salivary glands and saliva components has substantially increased in recent years because of high-throughput transcriptomic, proteomic, and functional analysis (33). Hundreds or thousands of salivary proteins have been identified that are capable of modulating vertebrate host defense by impairing inflammatory reaction, hemostasis, complement cascade, and immunity. Some have been functionally annotated and can be grouped into several protein families, such as lipocalins, glycine-rich proteins, metalloproteases, anticoagulants of the madanin family, and mucins.

### Metalloproteases

Tick metalloprotease proteins were identified in saliva, midgut and ovary and play a role in tick physiology, such as innate immunity, blood uptake, blood meal digestion, and vitellogenesis (34). Tick metalloproteases sequences from diverse tick species have been characterized, e.g., *I. scapularis, I. ricinus, Ornithodoros savignyi, R. sanguineus, R. microplus, A. americanum*, and *Amblyomma maculatum* (35). Putative metalloproteases (Metis 1 and Metis 2) were identified from the hard tick *I. ricinus*. RNAi-mediated gene silencing of Metis 1–2 induced a high mortality rate. Vaccination of rabbits with recombinant Metis 1 protein reduced both weight gain and oviposition rate of *I*. *ricinus*, but had no effect on mortality and feeding time of ticks (36). Immunization with another recombinant metalloprotease HLMP1 from the *Haemaphysalis longicornis* resulted in increased mortality up to 15% of ticks (37). The vaccine efficacy with recombinant metalloprotease BrRm-MP4 from the salivary glands of *R*. *microplus* was 60% (38). Due to these promising results, the therapeutic potential for metalloprotease proteins might be a challenging task due to their stability and the long-term maintenance of their activity (33).

### Protease Inhibitor Families

Several tick protease inhibitor families have been reported in the salivary glands, which have an important role in tick–host interactions (39). There are four groups under serine protease inhibitors, namely Kunitz, trypsin inhibitor-like domain, Kazal-domain inhibitors, and serpins, while the cysteine protease inhibitors belong to the cystatin family (40). The therapeutic potential of Kunitz-, Kazal-type, and TIL-domain inhibitors are reviewed elsewhere (40). Here, we discuss the vaccine potential of the other two groups, serine protease inhibitors and cystatins.

Serine proteinase inhibitors (SERPINs) are one of the largest and most abundant super family of proteinase inhibitors that play essential roles in every living system, from animals to plants. This enzyme family is conserved throughout evolution, and these proteins are involved in normal tick physiology, including the production of antimicrobial peptides, melanization, innate immunity, and blood digestion (41, 42). A flexible reactive center loop (RCL) of SERPINs with a conserved domain structure of 350–400 amino acid residues makes their characteristic structure (42). It has been shown that SERPINs differ from Kunitz-domain protease inhibitors by a distinctive conformational change of the RCL during the inhibition of their target proteases (43). SERPINs also have the ability to bind various protease and non-protease ligands, for example kallistatin, nexin-1, headpin, maspin, C1-inhibitor, and antichymotrypsin (44). SERPINs sequences from diverse tick species have been identified, including *Amblyomma hebraeum, Amblyomma variegatum, A. americanum, A. maculatum, Dermacentor variabilis, H. longicornis, R. appendiculatus, R. microplus, I. ricinus*, and *I. scapularis* (45). Despite high numbers of SERPINs from different tick species being identified in both *in vitro* and *in vivo* experimental analyses, only a few of them have been characterized functionally. IRIS (*I. ricinus* immunosuppressor), an immunomodulatory serpin from the salivary glands of *I. ricinus*, has an effect on host defense mechanisms by interfering with the production of pro-inflammatory cytokines (46). IRIS, together with a serpin from *H. longicornis* (HLS1) and several other proteins, have methionine and cysteine in their reactive center loop (RCL) and lack a signaling peptide, suggesting intracellular rather than extracellular function. Immunization of rabbits with recombinant IRIS showed significant protection (up to 30% mortality of feeding nymphs and adults) against *I. ricinus* infestation, but had no effect on nymphs fed on vaccinated mice (47). HLS2 was identified from *H. longicornis*, and contains a signal sequence and RCL with high sequence similarities to both invertebrate and vertebrate serpins. HLS2 also demonstrated significant protective immunity against adults and nymphs fed on immunized rabbits (48). Four serpins (RAS-1,2,3,4) were identified from *R. appendiculatus* (40). They were tested in a vaccine trial either as a mix of recombinant RAS-1 and RAS-2 or as a cocktail composed of three antigens, RAS-3, RAS-4, and a 36-kDa immunodominant cement protein RIM36. Vaccination of cattle with recombinant RAS-1 and RAS-2 cocktail reduced the number of engorged *R. appendiculatus* nymphs by 61 and a 43% and 28 increase in mortality rate in adult males and females, respectively. Similarly, a combination of RAS-3, RAS-4, and RIM36 resulted in a 40% mortality rate for *R. appendiculatus* ticks and almost 50% for *Theileria parva*-infected female ticks. Nevertheless, no significant protective effect against infection with *T. parva* was reported (49, 50).

The second-largest group of tick protease inhibitors is cystatins that are ubiquitously distributed in a wide variety of organisms. Tick cystatins either modulate vertebrate biological processes during tick feedings, such as immunity, antigen processing and presentation, phagocytosis, cytokine expression, and apoptosis, or can regulate hemoglobin digestion in ticks, which is driven by cathepsins (39). There are four cystatin subgroups based on conserved sequence motifs and cystatin domains: type 1 cystatins (stefins), type 2 cystatins, type 3 cystatins (kininogens), and type 4 cystatins (histidine-rich proteins, fetuins) (51). Among them, type 1 and 2 cystatins have been reported in ticks. The type 1 cystatins are typically intracellular and non-secreted cysteine protease inhibitors, while type 2 cystatins are secreted proteins that contain signal peptides as well as single cystatin-like domain. The type 1 (stefins) was the first cystatin isolated from *R. (Boophilus) microplus* that can inhibit tick vitellin degrading cysteine endopeptidase (VDTCE) and human cathepsin L, suggesting an immunomodulatory role and interaction with the vertebrate pro-inflammatory immune system (52). Similarly to serpins, several cystatins from different tick species have been reported such as Bmcystatin from *R. microplu*s, one cystatin from *A. americanum*, type 2 cystatin from *H. longicornis*, type 1 intracellular cystatin from *H*. *longicornis*, JpIocys2 from *Ixodes ovatus*, Om-cystatin 1 and 2 from *Ornithodoros moubata* soft tick and sialostatin L, L2 from *I. scapularis* (53). Sialostatin L2 and sialostatin L, novel type 2 secreted cysteine protease inhibitors, were biochemically and immunologically characterized from the salivary glands of *I. scapularis* ticks that are potentially implicated in the transmission of TBPs. Sialo L2 has been found to interfere with interferon-mediated immune responses in mouse dendritic cells (DC), resulting in enhanced replication of TBEV in bone marrow DC. Further, Sialo L2 promotes infection of mice by impairing inflammasome formation upon the presence of *A. phagocytophilum* in macrophages through targeting caspase-1 mediated inflammation, suggesting an important role in tick–pathogen interactions (54). Moreover, RNA silencing of sialo L2 resulted in increased mortality of ticks (up to 40%), as well as reduced tick size. Similar effects were reported when *I. scapularis* nymphs were exposed to guinea pigs immunized with sialo L2 (55). Several other cystatins from different tick species have been reported. Cystatin OmC2 was identified from the soft tick *O. moubata*. Immunization of mice with recombinant OmC2 significantly reduced the feeding ability and increased mortality (53). The vast majority of cystatin protease inhibitors identified in saliva have anti-inflammatory and immunosuppressive effects at the tick-host interface, suggesting cystatins might also be attractive anti-tick vaccine candidates. Targeting cysteine protease inhibitors does not only block tick attachment to the host and acquisition of the blood meal, but also impairs tick-borne pathogen transmission as demonstrated by vaccination of guinea pig against sialostatin L2 from *I. scapularis* (53, 55).

## Transmembrane Proteins

Vaccine efficacy of transmembrane proteins is well-established since Bm86-based vaccines in cattle tick *R*. *microplus* have shown to be an effective tick control strategy. It has been reported that global vaccine efficacies vary from 45 to 100% because of sequence heterogeneity in the Bm86 gene among geographically separated *R. microplus* strains. A study on Thai cattle ticks found that Bm86 sequences were 91–93% similar to the amino acid reference sequences (56). Nevertheless, it is still the best option available for controlling ticks. Studies with Bm95 (homolog of Bm86 from *R. microplus*) demonstrated that it protects against a wide range of cattle ticks from different localities, suggesting that the Bm95-based vaccine could be a more universal choice (57, 58). Similarly, vaccination with recombinant *Rhipicephalus annulatus* Ba86 (an ortholog of Bm86 from *R. microplus*) was protective against *R. annulatus* and *R. microplus* infestations. The vaccine efficacy of Ba86 was slightly higher for *R. annulatus* than for *R. microplus* as expected (59). A recombinant *Hyalomma anatolicum anatolicum* Haa86 antigen is a Bm86 ortholog that is not only effective against tick infestations but also decreased *Theileria annulata* transmission (60, 61). ATAQ, a new member of promising antigen has been identified from metastriate ticks with high sequence similarity with Bm86 which is expressed in the tick gut and Malpighi tubes, unlike Bm86 orthologs that are expressed primarily in tick midgut (62). This novel protein contains specific epidermal growth factor-like domains (EGF-like) that could be considered as multi-antigen vaccine combination with carriers and adjuvants (63). Apart from Bm86 orthologs and homologs, the only other published antigen with a membrane association was aquaporin. Tick aquaporins belong to the membrane intrinsic protein (MIP) superfamily which is known to play a key role in the transportation of water, glycerol, and urea across the cell membrane. Osmoregulation and water balance functions of aquaporins were examined in *I. ricinus* by RNAi, which significantly reduced the body weight of semi-engorged females that fed for 5 days. A reported 75% vaccination trial efficacy has been reported against *R. microplus* infestations (64). Transcriptome studies of *R. microplus* showed that RmAQP1 as a potential vaccine antigen has significant amino acid sequence similarity to the human aquaporin 7 family and bovine AQP1 orthologs (64). This challenge can potentially be overcome by identifying highly conserved peptide motifs among tick AQP1 that differ from bovine and human AQP1 (65). It should be further tested against different tick species in different geographical regions.

Transmembrane proteins (TM) prediction using bioinformatic methods are reliable because of their transmembrane topology, simple structural features, signal peptide, and membrane-spanning regions (66). Richards et al. (67) have identified 878 putative TM from *R. microplus* which contribute to our understanding of membrane proteins as a whole and the possibility of using the identified proteins as anti-tick vaccines. TM (as a protein family) have not yet been fully exploited in vaccination studies, though their conserved molecular functions in both prokaryotic and eukaryotic organisms, such as chemical communication between the cell and its environment, cell-cell interactions, metabolism, transport of metabolites through ion channels, signal transduction, and recognition (68). More than 50% of the marketed drugs are targeting peptidergic G-protein-coupled receptors (GPCRs), highlighting their potential pharmacological and biological roles. GPCRs, also called seven-transmembrane receptors, are the most intensively studied drug targets in human medicine. Thus, the discovery of a tick neuropeptide GPCR may have commercial potential for tick control as lead molecules for drug/vaccine development. Moreover, there are no true orthologous mammalian receptors of the tick kinin receptor, which makes it an attractive potential (69, 70).

## Blood Digestion Machinery in Ticks

The digestive tract in both ecto- and endoparasites is an excellent potential target for vaccine development, especially through the inhibition of blood digestion (71). It has been reasonably well-established that the tick digestive system is divided into three compartments, namely the foregut, midgut, and hindgut, and each part is further subdivided based on their function and location (72). Digestive cells of the tick midgut are playing an important role in blood digestion. Feeding and digestion of host blood are fundamental biological processes for ticks, which are essential for nutrition, development and transmission of pathogens. Unlike blood-feeding insects that digest blood in the neutral or alkaline pH of the gut lumen (73), ticks digest hemoglobin and other proteins intracellularly (within the endosomes of gut epithelium) in the acidic lysosome-like vesicles of digestive cells lining the midgut epithelium (74). There are notable differences in blood-feeding strategies between soft and hard ticks. Hard tick blood feeding lasts for several days or even longer followed by rapid engorgement (10–22 h), and feed only once in each active life-cycle stage. In contrast to hard ticks, soft ticks take their blood meal very rapidly (0.5–1 h), and oviposition and feeding of their females are repeated. Following blood feeding, midgut epithelium uses receptor-mediated endocytosis and fluid-phase endocytosis (RME) to take up blood meals from the gut lumen (75). Tick hemoglobinolytic systems are carried out by an acidic peptidase (optimum pH 3.5) that contains aspartic peptidases of the cathepsin D type, cysteine endo, and exopeptidases of the papain type (cathepsin C, B, and L) and asparaginyl endopeptidases of the legumain type (75). This multi-enzyme cascade and unique mechanism of activation and substrate binding have the potential for vaccine or drug discovery, as demonstrated by the results of RNAi-mediated silencing of individual peptidases in non-insect arthropods (75). For example, knockdown of cathepsin B (longipain) in *H. longicornis* by RNAi resulted in reduced blood digestion and transmission of *Babesia* spp. to the host. Similarly, the silencing of legumain gene (HlLgm1 and HlLgm2) in *H. longicornis* resulted in reduced oviposition rate, as well as reduced engorged-tick body weight (76).

**Figure 2:**
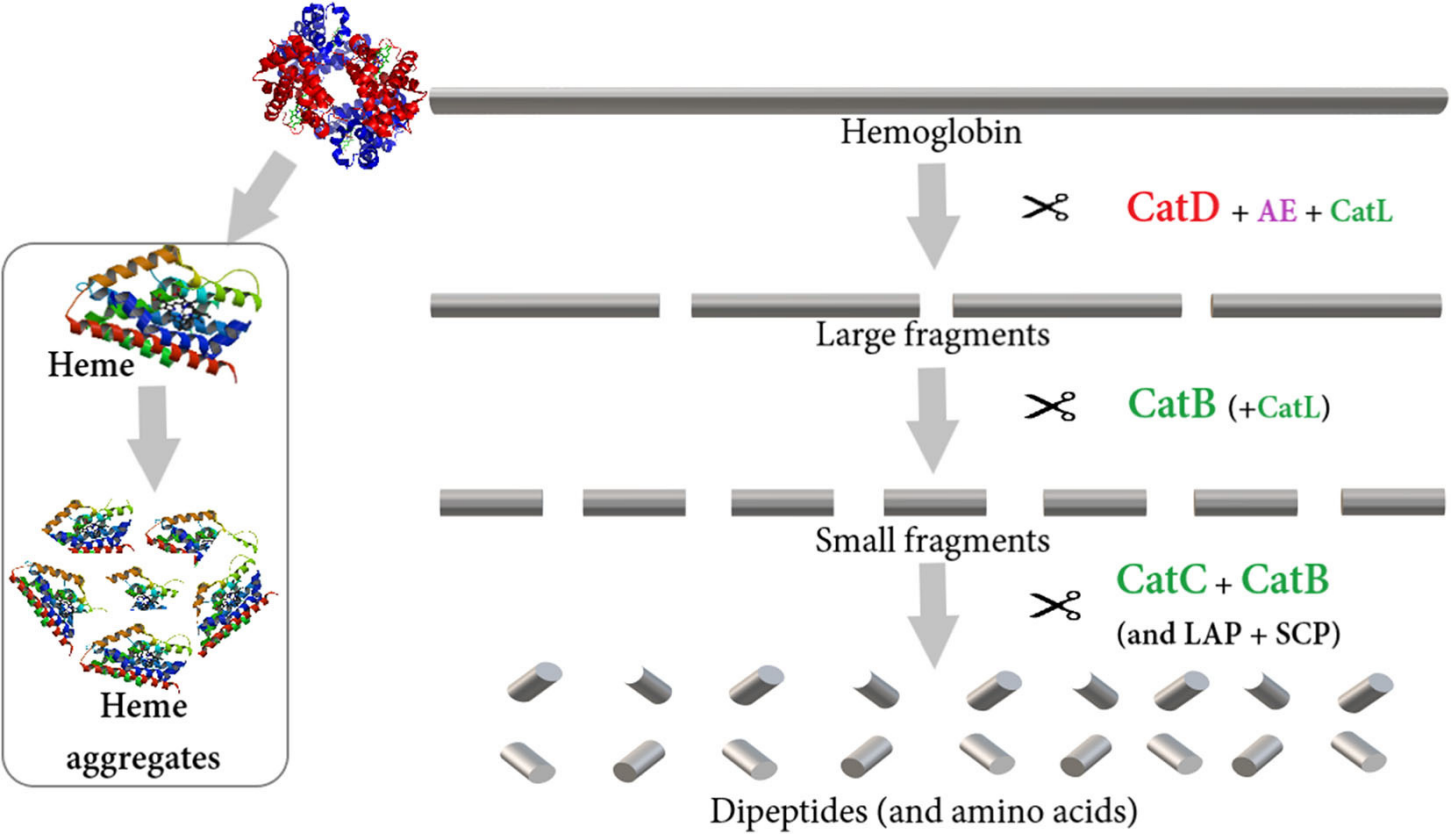
A proposed model for the hemoglobinolytic pathway in *I. ricinus* [modified from (75)]. The enzymes are color-coded according to clan membership- AA clan aspartic peptidases (red), CD clan cysteine peptidases (purple), CA clan (papain family) cysteine peptidases (green), and serine and metallopeptidases (black). The endopeptidases, cathepsin D (CatD) supported by cathepsin L (CatL) and legumain (AE), are responsible for the primary cleavage of hemoglobin. The production of secondary small fragments is dominated by the endopeptidase activity of cathepsin B (CatB). Exopeptidases act on the peptides released by the action of the endopeptidases through the carboxy-dipeptidase activity of CatB and the amino-dipeptidase activity of cathepsin C (CatC). Monopeptidases, including leucine aminopeptidase (LAP) and serine carboxypeptidase (SCP), might participate in the liberation of free amino acids.

The proposed hemoglobinolytic pathway was uncovered in the hard tick *I. ricinus* using proteomic analysis [(75); Figure 2]. Three endopeptidases are most likely responsible for gradual degradation of the hemoglobin substrate, namely cathepsin D (clan AA aspartic peptidases), cathepsin L (clan CA cysteine peptidases), and legumain (clan CD cysteine peptidases). Among these, aspartic cathepsin D is the most dynamic enzyme with high turnover efficiency. Subsequently, the cysteine proteases, namely cathepsin B (clan CA papain-family peptidase) and cathepsin C (clan CA dipeptidyl-peptidase) are the most abundant intestinal peptidases in the next phases of the hemoglobinolysis that cleave large hemoglobin fragments into dipeptides (75). Lists of cysteine and aspartic peptidases identified from different tick species are presented in Table 3.

**Table 3:** Lists of cysteine and aspartic peptidases identified from different tick species [modified from (75)].

**Tick species**	**Type of peptidases**	**Name of peptidase**	**Functional characterization**	**Tissue expression**
*Haemaphysalis longicornis*	Serine proteases	HlSP, HlSP2, HlSP3	RNAi and recombinant enzymes	Midgut and midgut lumen
*Haemaphysalis longicornis*	Legumain	H1Lgm, H1Lgm2	RNAi and recombinant enzymes	Midgut
*Ixodes ricinus*	Cathepsin L	IrCL1	RNAi and native or recombinant enzymes	Midgut, salivary glands, ovaries, and Malpighian tubules
*Rhipicephalus microplus*	Cathepsin L	BmCL1	Native or recombinant enzymes	Midgut
*Haemaphysalis longicornis*	Cathepsin B	Longipain	RNAi and recombinant enzymes	Midgut
*Ixodes ricinus*	Cathepsin D	IrCD1	RNAi and native or recombinant enzymes	Midgut
*Haemaphysalis longicornis*	Cathepsin D	Longepsin	RNAi and recombinant enzymes	Midgut and salivary glands
*Haemaphysalis longicornis*	Leucine amidopeptidase	HlLAP	RNAi and recombinant enzymes	Midgut, salivary glands, ovaries, and epidermis
*Ixodes ricinus*	Legumain	IrAE	RNAi and native or recombinant enzymes	Midgut
*Haemaphysalis longicornis*	Cathepsin L	H1CPL-A	Recombinant enzymes	Midgut
*Ixodes ricinus*	Cathepsin C	IrCC	Native enzyme	Midgut, salivary glands, ovaries, and Malpighian tubules
*Ixodes ricinus*	Cathepsin B	IrCB1	Native enzyme	Midgut
*Rhipicephalus microplus*	Cathepsin D	BmAP	Native enzyme	Midgut

A recent study showed that vaccination with cathepsin D has the potential to control poultry red mite infestations of chickens (77). However, vaccination trials on animals with an individual recombinant digestive enzyme, along with a cocktail of recombinant antigens, did not induce any significant level of protection against *I. ricinus* (78). Because of aspartic peptidases (APDs) redundancy in tick digestive apparatus, a multi-enzyme target strategy (a combination of aspartic and cysteine protease) might be necessary to exploit tick digestive hemoglobinolytic enzymes as new candidate antigens for the development of tick vaccines. It has been proposed that parasite vaccines should be based on at least two enzymes involved in different physiological processes, for example, blood digestive protein, together with proteins involved in essential physiological processes of ticks. As such, two combined recombinant hookworm antigens, glutathione S-transferases (GSTs) and cathepsin D-like aspartic protease haemoglobinase (APR-1), eventually became a leading vaccine candidate against the human hookworm *Necator americanus* (79). In this regard, a multi-antigenic vaccine against *R. microplus* was tested on cattle under field conditions. Vaccination studies with recombinant *Boophilus* yolk pro-cathepsin (BYC) and vitellogenin degrading cysteine endopeptidase (VTDCE) from *R*. *microplus*, and Hl-GST from *H. longicornis* affect tick physiology, decrease the number of semi-engorged female ticks in the host, resulting in an improved body weight gain of cattle (80). Similarly, cathepsin L and cathepsin D both have potential as vaccine antigens as part of a multi-component vaccine for controlling the poultry red mite *Dermanyssus gallinae* (81). Future work should be focused on identifying the appropriate synergistic combinations of these proteins to develop a cocktail vaccine for novel therapeutic interventions.

## Iron Acquisition and Metabolism

During blood feeding, ticks have to protect themselves from the toxicity of heme and iron from the host blood. Free iron radicals are potentially toxic for all organisms that cause substantial damage to lipids, DNA and protein molecules (82). Unlike ticks, iron originated from heme degradation in hematophagous insects is catalyzed by heme oxygenase (HO) (83). This trait is, however, absent from other blood feeding non-insect arthropods like ticks and mites. Ticks possess a complex biochemical process via hemosome formation that efficiently utilizes non-heme iron within the tick body. Three proteins are involved in vertebrate iron metabolism, namely ferritin (Fer) and the iron regulatory proteins (IRP1) and IRP2 (84). Intracellular ferritin (Fer1) was identified in both soft and hard ticks, which shared sequence similarity with mammalian heavy-chain ferritins and conserved ferroxidase sites (85). Afterward, Fer2 and iron-responsive protein (IRP) were described in *I*. *ricinus*, which are exclusively expressed in tick gut, and are required for iron transport to the peripheral tissues for their normal function (84). It has been shown that Fer2 possesses all the important characteristics for the development of a vaccine, including: not being presented in the vertebrate host (concealed antigen property), non-redundancy gene, low homology to mammalian ferritins, and host antibodies can directly contact the tick gut while feeding on a vaccinated host (86). A study showed that vaccination with recombinant tick Fer2 has the potential to control tick infestations on rabbits against *I. ricinus* (87) and *H. longicornis* (88). Essentially, the vaccine efficacy of recombinant Fer2 from *R. microplus* was comparable with the commercial vaccine based on the Bm86 antigen (87). Furthermore, GSTs are present in ticks where they serve primarily in biological detoxification. This enzyme is capable of binding heme in blood-feeding arthropods including ticks, suggesting that GSTs can serve as detoxifying molecules. A vaccination trial on bovines with recombinant GST from *H. longicornis* showed a cross-protection immunity against infestation with *R. microplus* (89).

## Reverse Vaccinology Approaches in Antigen Discovery

According to Louis Pasteur's principle, vaccine development was based on the “isolate–inactivate–inject” approach before the 1990s. The “first generation” vaccine included live, attenuated and killed types, while the “second generation” vaccine consisted of purified or recombinant components of the targeted antigen. Nevertheless, this approach is a long and laborious task even in successful projects. Besides, these approaches are often unsuccessful because of the complexity of tick's life cycle, complicated host-pathogen-tick interactions and pathogens not possessing clear immunogenic properties. Thus, next-generation vaccination approaches might overcome these challenges by using a combination of functional genomics, bioinformatics and systems biology methodologies (90). This method involves *in silico* screening of the whole genome of a tick to identify genes with important immunogenic properties followed by wet lab verification with vaccination trials. Tick antigens developed based on biological processes and vaccine approaches tested for vaccine development against ticks are listed in Table 4 (91–95).

**Table 4:** Tick antigens developed based on biological process and vaccine approaches tested for vaccine development against ticks.

**Protein type**	**Species**	**Identity of protein**	**Antigen**	**Vaccination approach**	**Efficacy of vaccine**	**References**
Secreted	*Rhipicephalus appendiculatus, R. sanguineus*	Putative cement protein	64TRP	Recombinant protein, 2nd generation	62 and 47% mortality	(21, 22)
	*Rhipicephalus appendiculatus*	Serine protease inhibitor	RAS-3, RAS-4, RIM36	Recombinant protein, 2nd generation	39 and 48% mortality	(49, 50)
	*Ixodes scapularis*	Cystatin type 2	Sialostatin L2	Recombinant protein, 2nd generation	40% mortality	(55)
	*Ixodes scapularis*	Cystatin type 2	Sialostatin L	Recombinant protein, 2nd generation	Not reported	(55)
	*Ixodes ricinus*	Serine protease inhibitor	IRIS	Recombinant protein, 2nd generation	30% mortality	(47)
	*Rhipicephalus microplus, R. annulatus*	Ferritin, iron transporter	RaFER2/RmFER2	Recombinant protein, 2nd generation	64 and 72% efficacy	(87)
Membrane associated	*Rhipicephalus microplus*	Angiotensin converting enzyme	Bm91	Recombinant protein, 2nd generation	6% reduction of reproductive index	(91)
	*Rhipicephalus microplus, R. annulatus*	Aquaporin	Aquaporin	Reverse vaccinology, 3rd generation	75 and 68% efficacy	(64, 65)
	*Rhipicephalus appendiculatus, R. decoloratus, R. microplus, Hyalomma anatolicum, H. dromedarii*	Bm86 and orthologs	Bm86 and orthologs	Recombinant protein, 2nd generation	45–100% efficacy	(57–61)
	*Rhipicephalus microplus*	5′-nucleotidase	4F8	Recombinant protein, 2nd generation	No efficacy	(92)
	*Rhipicephalus microplus*	Mucin	BMA7	Purified components, 2nd generation	21% reduction in egg weights	(93)
Intracellular	*Rhipicephalus microplus*	Elongation factor	Ef1a	Recombinant protein, 2nd generation	31% efficacy	(94)
	*Rhipicephalus sanguineus, R. microplus*	Acidic ribosomal protein P0	pP0	Synthetic peptide, 2nd generation	96% efficacy	(95)
	*Rhipicephalus microplus*	Glutathione S transferase	GST-HI	Recombinant protein, 2nd generation	57% efficacy	(89)
	*Rhipicephalus annulatus, R. microplus*	Regulator factor	Subolesin (4D8)	Recombinant protein, 2nd generation	0–83% efficacy	(19, 20, 23)
	*Rhipicephalus microplus, R. annulatus*	Ubiquitin	UBE	Recombinant protein, 2nd generation	15 and 55% efficacy	(94)

**Figure 3:**
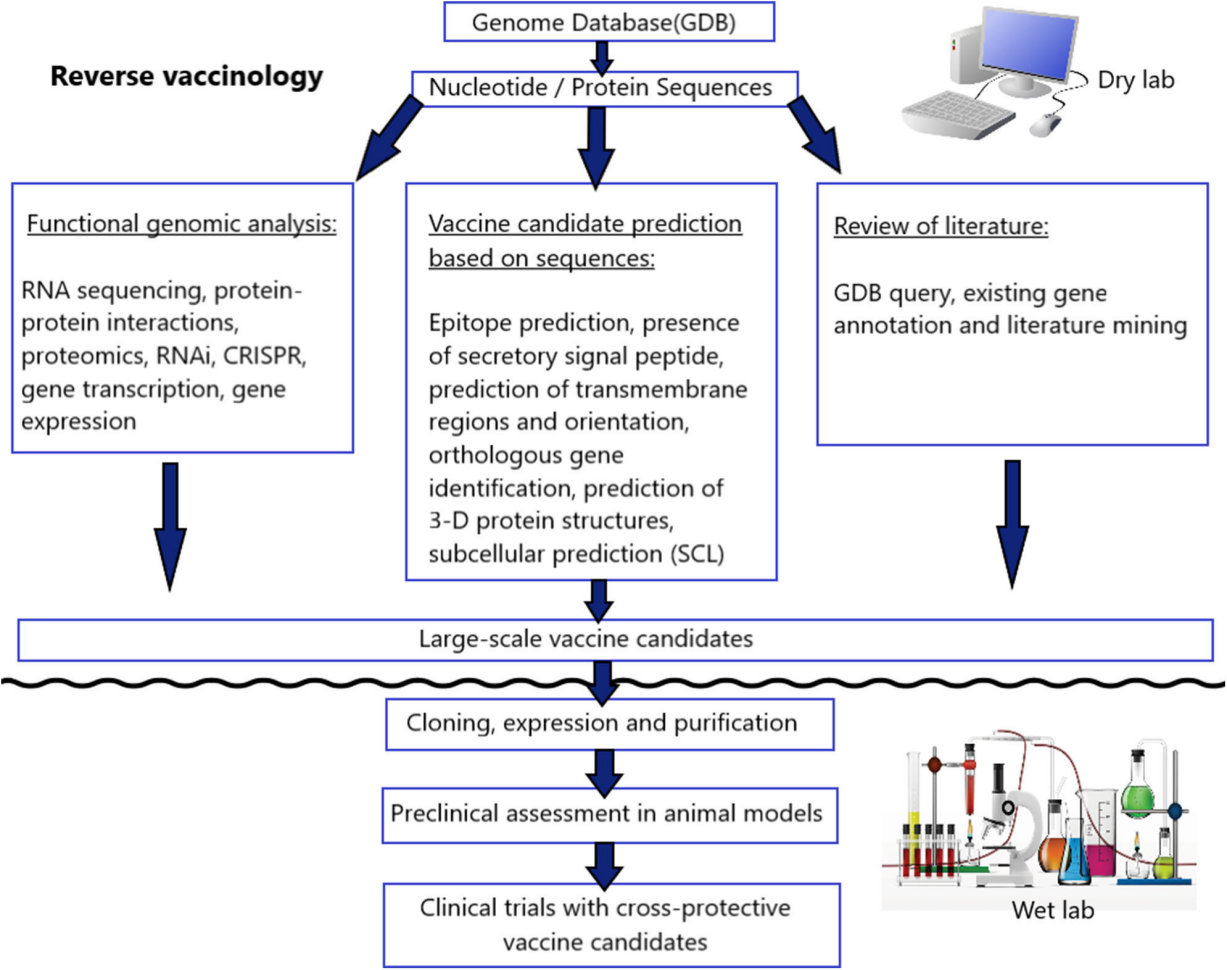
A schematic representation of the integrative reverse vaccinology approach toward vaccine development.

Reverse Vaccinology (RV) is a new methodology to design vaccines from systems biology and functional genomic information with the help of bioinformatics, which was first described in the year 2000 [(96); Figure 3]. Since approximately 2005, two types of RV approaches have been developed for the anti-tick vaccine study, one involving the use of gene knockdown study (RNAi) and the other involving the use of microarray analysis, EST library, subtraction libraries, and CattleTickBase approaches (97). RNAi has the potential to improve understanding of gene function where functional genomic data are limited. It has been reported in several tick species, namely *Rhipicephalus haemaphysaloides, A. americanum, H. longicornis, I. scapularis*, and *D. variabilis*. Another RV technique was developed based on a “systems biology” analysis of transcriptome datasets and RNAi screening of candidates, which identified aquaporin antigen from *R. microplus* (64, 98). Functional genomics has been applied to study the interaction of tick-pathogen-host for transmission blocking vaccine development. For example, molecular studies have been conducted to study the pathogen transmission at the tick-*Anaplasma* interface, either *in vitro* or *in vivo* (99). However, gene knockdown study is slow throughput, off-target effect, difficult to find an appropriate delivery system, the incompleteness of knockdowns and time-consuming approach (100). Hence, an alternative gene-manipulation approach such as CRISPR-based gene drive could be explored for the production of more efficient, stable, and safe parasite vaccines (101, 102).

## Conclusions

This review highlights the progress, opportunities, and challenges offered by recent research into vaccination, as well as provide a wealth of unprecedented information on the genes and proteins involved in the blood digestion process in the ticks, tick-host-pathogen interface, and proteins involved in pathogen transmissions, along with the discussion of potential vaccine targets in recent years. Based on our growing knowledge of tick genome complexity and protein superfamilies, tick vaccines are entering a new genomic era that will facilitate the discovery of novel vaccine antigens. It is becoming clear that the “One Health” approach is needed for developing anti-tick vaccines which means multidisciplinary sectors (biologists, immunologists, bioinformaticians, physicians, veterinarians and pharmaceutical industry, amongst others) communicate and work together to achieve better outcomes.

## Author Contributions

QH and BB conceived and designed the study protocol and participated in the writing of the manuscript. BB was a major contributor in writing the manuscript. This manuscript was approved by BB and QH.

## Conflict of Interest

The authors declare that the research was conducted in the absence of any commercial or financial relationships that could be construed as a potential conflict of interest.

## References

[1] Bhowmick B Zhao J Øines Ø Bi T Liao C Zhang L. Molecular characterization and genetic diversity of *Ornithonyssus sylviarum* in chickens (*Gallus gallus*) from Hainan Island, China. Par Vect. (2019) 12:553. 10.1186/s13071-019-3809-9 31753001 PMC6873570

[2] Jeyaprakash A Hoy MA. First divergence time estimate of spiders, scorpions, mites and ticks (subphylum: Chelicerata) inferred from mitochondrial phylogeny. Exp Appl Acarol. (2009) 47:1–18. 10.1007/s10493-008-9203-5 18931924

[3] de La Fuente J Estrada-Peña A Venzal J. Overview: ticks as vectors of pathogens that cause disease in humans and animals. Front Biosci. (2008) 13:6938–46. 10.2741/3200 18508706

[4] de la Fuente J Contreras M Estrada-Peña A Cabezas-Cruz A. Targeting a global health problem: vaccine design and challenges for the control of tick-borne diseases. Vaccine. (2017) 35:5089–94. 10.1016/j.vaccine.2017.07.097 28780117

[5] Kocan KM de la Fuente J Blouin EF Coetzee JF Ewing SA. The natural history of *Anaplasma marginale*. Vet Parasitol. (2010) 167:95–107. 10.1016/j.vetpar.2009.09.012 19811876

[6] Baneth G. Tick-borne infections of animals and humans: a common ground. Int J Parasitol. (2014) 44:591–6. 10.1016/j.ijpara.2014.03.011 24846527

[7] de la Fuente J Villar M Contreras M Moreno-Cid JA Merino O Pérez de la Lastra JM. Prospects for vaccination against the ticks of pets and the potential impact on pathogen transmission. Vet Parasitol. (2015) 208:26–9. 10.1016/j.vetpar.2014.12.015 25555312

[8] de Castro JJ. Sustainable tick and tick borne disease control in livestock improvement in developing countries. Vet Parasitol. (1997) 71:77–97. 10.1016/s0304-4017(98)00096-x 9261972

[9] Abbas R Zaman M Colwell D. Acaricide resistance in cattle ticks and approaches to its management: the state of play. Vet Parasitol. (2014) 203:6–20. 10.1016/j.vetpar.2014.03.006 24709006

[10] de la Fuente J Contreras M. Tick vaccines: current status and future directions. Expert Rev Vaccines. (2015) 14:1367–76. 10.1586/14760584.2015.1076339 26289976

[11] de la Fuente J Almazan C Canales M Perez de la Lastra JM Kocan KM Willadsen P. A ten-year review of commercial vaccine performance for control of tick infestations on cattle. Anim Health Res Rev. (2007) 8:23–8. 10.1017/S1466252307001193 17692140

[12] Garcia-Garcia JC Gonzalez IL Gonzalez DM Valdes M Mendez L Lamberti J. Sequence variations in the *Boophilus microplus* Bm86 locus andimplications for immunoprotection in cattle vaccinated with this antigen. Exp Appl Acarol. (1999) 23:883–95. 10.1023/a:1006270615158 10668863

[13] Nuttall PA Labuda M. Saliva-assisted transmission of tick-borne pathogens. In: Bowman AS Nuttall PA, editors. Ticks: Biology, Disease and Control. Cambridge: Cambridge University Press (2008) p. 205–19. 10.1017/CBO9780511551802.011

[14] Schuijt TJ Coumou J Narasimhan S Dai J Deponte K Wouters D. A tick mannose-binding lectin inhibitor interferes with the vertebrate complement cascade to enhance transmission of the lyme disease agent. Cell Host Microbe. (2011) 10:136–46. 10.1016/j.chom.2011.06.010 21843870 PMC3170916

[15] Dai J Narasimhan S Zhang L Liu L Wang P Fikrig E. Tick histamine release factor is critical for *Ixodes scapularis* engorgement and transmission of the Lyme disease agent. Plos Pathog. (2010) 6:e1001205. 10.1371/journal.ppat.1001205 21124826 PMC2991271

[16] Ramamoorthi N Narasimhan S Pal U Bao F Yang XF Fish D. The Lyme disease agent exploits a tick protein to infect the mammalian host. Nature. (2005) 436:573–7. 10.1038/nature03812 16049492 PMC4306560

[17] Narasimhan S Sukumaran B Bozdogan U Thomas V Liang X Deponte K. A tick antioxidant facilitates the Lyme disease agent's successful migration from the mammalian host to the arthropod vector. Cell Host Microbe. (2007) 2:7–18. 10.1016/j.chom.2007.06.001 18005713 PMC2699493

[18] Sukumaran B Narasimhan S Anderson JF DePonte K Marcantonio N Krishnan MN. An *Ixodes scapularis* protein required for survival of *Anaplasma phagocytophilum* in tick salivary glands. J Exp Med. (2006) 203:1507–17. 10.1084/jem.20060208 16717118 PMC2118316

[19] Bensaci M Bhattacharya D Clark R Hu LT. Oral vaccination with vaccinia virus expressing the tick antigen subolesin inhibits tick feeding and transmission of *Borrelia burgdorferi*. Vaccine. (2012) 30:6040–6. 10.1016/j.vaccine.2012.07.053 22864146 PMC3938954

[20] Merino O Almazán C Canales M Villar M Moreno-Cid JA Galindo RC. Targeting the tick protective antigen subolesin reduces vector infestations and pathogen infection *by Anaplasma marginale* and *Babesia bigemina*. Vaccine. (2011) 29:8575–9. 10.1016/j.vaccine.2011.09.023 21951878

[21] Rego ROM Trentelman JJA Anguita J Nijhof AM Sprong H Klempa B. Counterattacking the tick bite: towards a rational design of anti-tick vaccines targeting pathogen transmission. Parasit Vectors. (2019) 12:229. 10.1186/s13071-019-3468-x 31088506 PMC6518728

[22] Labuda M Trimnell AR Licková M Kazimírová M Davies GM Lissina O. An anti-vector vaccine protects against a lethal vector-borne pathogen. PLoS Pathog. (2006) 2:e27. 10.1371/journal.ppat.0020027 16604154 PMC1424664

[23] Havlíková S Ličková M Ayllón N Roller L Kazimírová M Slovák M. Immunization with recombinant subolesin does not reduce tick infection with tick-borne encephalitis virus nor protect mice against disease. Vaccine. (2013) 31:1582–9. 10.1016/j.vaccine.2013.01.017 23357197

[24] Antunes S Merino O Mosqueda J Moreno-Cid JA Bell-Sakyi L Fragkoudis R. Tick capillary feeding for the study of proteins involved in tick-pathogen interactions as potential antigens for the control of tick infestation and pathogen infection. Parasit Vectors. (2014) 7:42. 10.1186/1756-3305-7-42 24450836 PMC3900739

[25] Juncadella IJ Anguita J. The immunosuppresive tick salivary protein, Salp15. Adv Exp Med Biol. (2009) 666:121–31. 10.1007/978-1-4419-1601-3_10 20054980

[26] Dai J Wang P Adusumilli S Booth CJ Narasimhan S Anguita J. Antibodies against a tick protein, Salp15, protect mice from the Lyme disease agent. Cell Host Microbe. (2009) 6:482–92. 10.1016/j.chom.2009.10.006 19917502 PMC2843562

[27] Nuttall PA Tick saliva and its role in pathogen transmission. Wien Klin Wochenschr. (2019). 10.1007/s00508-019-1500-y [Epub ahead of print]. PMC10118219 31062185

[28] Sultana H Neelakanta G Kantor FS Malawista SE Fish D Montgomery RR. *Anaplasma phagocytophilum* induces actin phosphorylation to selectively regulate gene transcription in *Ixodes scapularis* ticks. J Exp Med. (2010) 207:1727–43. 10.1084/jem.20100276 20660616 PMC2916137

[29] Liu L Narasimhan S Dai J Zhang L Cheng G Fikrig E. *Ixodes scapularis* salivary gland protein P11 facilitates migration of *Anaplasma phagocytophilum* from the tick gut to salivary glands. EMBO Rep. (2011) 12:1196–203. 10.1038/embor.2011.177 21921936 PMC3207102

[30] Paesen GC Adams PL Harlos K Nuttall PA Stuart DI. Tick histamine-binding proteins: isolation, cloning, and three-dimensional structure. Molecular Cell. (1999) 3:661–71. 10.1016/s1097-2765(00)80359-7 10360182

[31] Mulenga A Macaluso KR Simser JA Azad AF. The American dog tick, *Dermacentor variabilis*, encodes a functional histamine release factor homolog. Insect Biochem Mol Biol. (2003) 33:911–9. 10.1016/s0965-1748(03)00097-3 12915182

[32] Bakshi M Kim TK Porter L Mwangi W Mulenga A. *Amblyomma americanum* ticks utilizes countervailing pro and anti-inflammatory proteins to evade host defense. PLoS Pathog. (2019) 15:e1008128. 10.1371/journal.ppat.1008128 31756216 PMC6897422

[33] Chmela?r J Kotál J Kova?ríková A Kotsyfakis M The use of tick salivary proteins as novel therapeutics. Front Physiol. (2019) 10:812 10.3389/fphys.2019.00812 31297067 PMC6607933

[34] Francischetti IM Mather TN Ribeiro JM. Cloning of a salivary gland metalloprotease and characterization of gelatinase and fibrin(ogen)lytic activities in the saliva of the Lyme disease tick vector *Ixodes scapularis*. Biochem Biophys Res Commun. (2003) 305:869–75. 10.1016/s0006-291x(03)00857-x 12767911 PMC2903890

[35] Ali A khan S Ali I Karim S da Silva Vaz I Jr Termignoni C Probing the functional role of tick metalloproteases *Physiol Entomol*. (2015) 40:177–88. 10.1111/phen.12104

[36] Decrem Y Beaufays J Blasioli V Lahaye K Brossard M Vanhamme L. A family of putative metalloproteases in the salivary glands of the tick *Ixodes ricinus*. FEBS J. (2008) 275:1485–99. 10.1111/j.1742-4658.2008.06308.x 18279375

[37] Imamura S da Silva VI Konnai S Yamada S Nakajima C Onuma M. Effect of vaccination with a recombinant metalloprotease from *Haemaphysalis longicornis*. Exp Appl Acarol. (2009) 48:345–58. 10.1007/s10493-009-9245-3 19184465

[38] Ali A Fernando Parizi L Garcia Guizzo M Tirloni L Seixas A da Silva Vaz I Jr. Immunoprotective potential of a *Rhipicephalus* (*Boophilus*) *microplus* metalloprotease. Vet Parasitol. (2015) 207:107–14. 10.1016/j.vetpar.2014.11.007 25480468

[39] Chmelar J Kotál J Karim S Kopacek P Francischetti IMB Pedra JHF. Sialomes and mialomes: a systems-biology view of tick tissues and tick–host interactions. Trends Parasitol. (2016) 32:242–54. 10.1016/j.pt.2015.10.002 26520005 PMC4767689

[40] Parizi LF Ali A Tirloni L Oldiges DP Sabadin GA Coutinho ML. Peptidase inhibitors in tick physiology. Med Vet Entomol. (2018) 32:129–44. 10.1111/mve.12276 29111611

[41] Meekins DA Kanost MR Michel K. Serpins in arthropod biology. Semin. Cell Dev Biol. (2017) 62:105–19. 10.1016/j.semcdb.2016.09.001 27603121 PMC5318264

[42] Potempa J Korzus E Travis J. The serpin superfamily of proteinase inhibitors: structure, function, and regulation. J Biol Chem. (1994) 269:15957–60. 8206889

[43] Rodriguez-Valle M Xu T Kurscheid S Lew-Tabor AE. Rhipicephalus microplus serine protease inhibitor family: annotation, expression and functional characterisation assessment. Parasit Vectors. (2015) 8:7. 10.1186/s13071-014-0605-4 25564202 PMC4322644

[44] Patston PA Church FC Olson ST. Serpin-ligand interactions. Methods. (2004) 32:93–109. 10.1016/S1046-2023(03)00201 14698622

[45] Ribeiro JM Anderson JM Manoukis NC Meng Z Francischetti IM. A further insight into the sialome of the tropical bont tick, *Amblyomma variegatum*. BMC Genomics. (2011) 12:136. 10.1186/1471-2164-12-136 21362191 PMC3060141

[46] Leboulle G Crippa M Decrem Y Mejri N Brossard M Bollen A. Characterization of a novel salivary immunosuppressive protein from *Ixodes ricinus* ticks. J. Biol. Chem. (2002) 277:10083–9. 10.1074/jbc.M111391200 11792703

[47] Prevot PP Couvreur B Denis V Brossard M Vanhamme L Godfroid E. Protective immunity against *Ixodes ricinus* induced by a salivary serpin. Vaccine. (2007) 25:3284–92 10.1016/j.vaccine.2007.01.008 17270322

[48] Imamura S Da Silva Vaz Junior I Sugino M Ohashi K Onuma M. A serine protease inhibitor (serpin) from *Haemaphysalis longicornis* as an anti-tick vaccine. Vaccine. (2005) 23:1301–11. 10.1016/j.vaccine.2004.08.041 15652673

[49] Imamura S Namangala B Tajima T Tembo ME Yasuda J Ohashi K. Two serine protease inhibitors (serpins) that induce a bovine protective immune response against *Rhipicephalus appendiculatus* ticks. Vaccine. (2006) 24:2230–7. 10.1016/j.vaccine.2005.10.055 16314008

[50] Imamura S Konnai S Vaz Ida S Yamada S Nakajima C Ito Y. Effects of anti-tick cocktail vaccine against *Rhipicephalus appendiculatus*. Jpn J Vet Res. (2008) 56:85–98. 10.14943/jjvr.56.2.85 18828446

[51] Rawlings ND Barrett AJ. Evolution of proteins of the cystatin superfamily. J Mol Evol. (1990) 30:60–71. 10.1007/bf02102453 2107324

[52] Schwarz A Valdés JJ Kotsyfakis M. The role of cystatins in tick physiology and blood feeding. Ticks Tick Borne Dis. (2012) 3:117–27. 10.1016/j.ttbdis.2012.03.004 22647711 PMC3412902

[53] Chmelar J Kotál J Langhansová H Kotsyfakis M. Protease inhibitors in tick saliva: the role of serpins and cystatins in tick-host-pathogen interaction. Front Cell Infect Microbiol. (2017) 7:216. 10.3389/fcimb.2017.00216 28611951 PMC5447049

[54] Chen G Wang X Severo MS Sakhon OS Sohail M Brown LJ. The tick salivary protein sialostatin L2 inhibits caspase-1-mediated inflammation during *Anaplasma phagocytophilum* infection. Infect Immun. (2014) 82:2553–64. 10.1128/IAI.01679-14 24686067 PMC4019176

[55] Kotsyfakis M Anderson JM Andersen JF Calvo E Francischetti IMB Mather TN. Cutting edge: immunity against a “silent” salivary antigen of the Lyme vector *Ixodes scapularis* impairs its ability to feed. J Immunol. (2008) 181:5209–12. 10.4049/jimmunol.181.8.5209 18832673 PMC2562228

[56] Kaewmongkol S Kaewmongkol G Inthong N Lakkitjaroen N Sirinarumitr T Berry CM. Variation among Bm86 sequences in *Rhipicephalus* (*Boophilus*) *microplus* ticks collected from cattle across Thailand. Exp Appl Acarol. (2015) 66:247–56. 10.1007/s10493-015-9897-0 25777941

[57] García-García JC Montero C Redondo M Vargas M Canales M Boué O. Control of ticks resistant to immunization with Bm86 in cattle vaccinated with the recombinant antigen Bm95 isolated from the cattle tick, *Boophilus microplus*. Vaccine. (2000) 18:2275–87. 10.1016/S0264-410X(99)00548-4 10717348

[58] de la Fuente J Kocan KM. Advances in the identification and characterization of protective antigens for development of recombinant vaccines against tick infestations. Exp Rev Vaccines. (2003) 2:583–93. 10.1586/14760584.2.4.583 14711342

[59] Canales M Almazán C Naranjo V Jongejan F delaFuente J. Vaccination with recombinant *Boophilus annulatus* Bm86 ortholog protein, Ba86, protects cattle against *B. annulatus* and *B. microplus infestations*. BMC Biotechnol. (2009) 9:29. 10.1186/1472-6750-9-29 19335900 PMC2667501

[60] Jeyabal L Azhahianambi P Susitha K Ray DD Chaudhuri P Hmuaka V. Efficacy of rHaa86, an orthologue of Bm86, against challenge infestations of *Hyalomma anatolicum anatolicum*. Transbound. Emerg. Dis. (2010) 57:96–102. 10.1111/j.1865-1682.2010.01107.x 20537118

[61] Azhahianambi P delaFuente J Suryanarayana VV Ghosh S Cloning, expression and immuno protective efficacy of rHaa86, the homologue of the Bm86 tick vaccine antigen, from *Hyalomma anatolicum anatolicum*. Parasite Immunol. (2009) 31:111–22. 10.1111/j.1365-3024.2008.01082.x 19222782

[62] Nijhof AM Balk JA Postigo M Rhebergen AM Taoufik A Jongejan F. Bm86 homologues and novel ATAQ proteins with multiple epidermal growth factor (EGF)-like domains from hard and soft ticks. Int J Parasitol. (2010) 40:1587–97. 10.1016/j.ijpara.2010.06.003 20647015 PMC2998001

[63] Aguirre AAR Lobo FP Cunha RC Garcia MV Andreotti R. Design of the ATAQ peptide and its evaluation as an immunogen to develop a *Rhipicephalus* vaccine. Vet Parasitol. (2016) 221:30–8. 10.1016/j.vetpar.2016.02.032 27084468

[64] Guerrero FD Andreotti R Bendele KG Cunha RC Miller RJ Yeater K. *Rhipicephalus* (*Boophilus*) *microplus* aquaporin as an effective vaccine antigen to protect against cattle tick infestations. Parasit Vectors. (2014) 7:475 10.1186/s13071-014-0475-9 25306139 PMC4200143

[65] Ndekezi C Nkamwesiga J Ochwo S Kimuda MP Mwiine FN Tweyongyere R. Identification of Ixodid tick-specific aquaporin-1 potential anti-tick vaccine epitopes: an *in-silico* analysis. Front. Bioeng. Biotechnol. (2019) 7:236. 10.3389/fbioe.2019.00236 31612130 PMC6775757

[66] Chen YK Li KB. Predicting membrane protein types by incorporating protein topology, domains, signal peptides, and physicochemical properties into the general form of Chou's pseudo amino acid composition. J Theor Biol. (2013) 318:1–12. 10.1016/j.jtbi.2012.10.033 23137835

[67] Richards SA Stutzer C Anna-Mari Bosman AM Maritz-Olivier C. Transmembrane proteins – mining the cattle tick transcriptome. Ticks Tick Borne Dis. (2015) 6:695–710. 10.1016/j.ttbdis.2015.06.002 26096851

[68] Hubert P Sawma P Duneau JP Khao J Hénin J Bagnard D. Single-spanning transmembrane domains in cell growth and cell-cell interactions. Cell Adh Migr. (2010) 4:313–24. 10.4161/cam.4.2.12430 20543559 PMC2900628

[69] Bhowmick B Tang Y Lin F Øines Ø Zhao J Liao C Comparative morphological and transcriptomic analyses reveal novel chemosensory genes in the poultry red mite, *Dermanyssus gallinae* and knockdown by RNA interference. bioRxiv [preprint]. (2020). 10.1101/2020.04.09.034587 PMC7578799 33087814

[70] Xiong C Baker D Pietrantonio PV. The cattle tick, *Rhipicephalus microplus*, as a model for forward pharmacology to elucidate Kinin GPCR function in the Acari. Front Physiol. (2019) 10:1008. 10.3389/fphys.2019.01008 31447698 PMC6692460

[71] Willadsen P. Novel vaccines for ectoparasites. Vet Parasitol. (1997) 71:209–22. 10.1016/S0304-4017(97)00028-9 9261979

[72] Pritchard J Kuster T Sparagano O Tomley F. Understanding the biology and control of the poultry red mite *Dermanyssus gallinae*: a review. Avian Pathol. (2015) 44:143–53. 10.1080/03079457.2015.1030589 25895578

[73] Briegel H Lea AO. Relationship between protein and proteolytic activity in the midgut of mosquitoes. J Insect Physiol. (1975) 21:1597–604. 10.1016/0022-1910(75)90197-3 240894

[74] Grandjean O. Aeschlimann A. Contribution to the study of digestion in ticks: histology and fine structure of the midgut ephithelium of *Ornithodorus moubata*, Murray (Ixodoidea, Argasidae). Acta Trop. (1973) 30:193–212 4147871

[75] Sojka D Franta Z Horn M Caffrey CR Mares M Kopacek P. New insights into the machinery of blood digestion by ticks. Trends Parasitol. (2013) 29:276–85. 10.1016/j.pt.2013.04.002 23664173

[76] Tsuji N Miyoshi T Battsetseg B Matsuo T Xuan X Fujisaki K. A cysteine protease is critical for *Babesia* spp. transmission in Haemaphysalis ticks. PLoS Pathog. (2008) 4:e1000062. 10.1371/journal.ppat.1000062 18483546 PMC2358973

[77] Price DRG Küster T Øines Ø Margaret Oliver E Bartley K Nunn F. Evaluation of vaccine delivery systems for inducing long-lived antibody responses to *Dermanyssus gallinae* antigen in laying hens. Avian Pathol. (2019) 48:S60–74. 10.1080/03079457.2019.1612514 31032631

[78] de la Fuente J Kopáček P Lew-Tabor A Maritz-Olivier C. Strategies for new and improved vaccines against ticks and tick-borne diseases. Parasite Immunol. (2016) 38:754–69. 10.1111/pim.12339 27203187

[79] Hotez PJ Diemert D Bacon KM Beaumier C Bethony JM Bottazzi ME. The human hookworm vaccine. Vaccine. (2013) 31:B227–32. 10.1016/j.vaccine.2012.11.034 23598487 PMC3988917

[80] Seixas A Oliveira P Termignoni C Logullo C Masuda A. *Rhipicephalus (Boophilus*) *microplus* embryo proteins as target for tick vaccine. Vet Immunol Immunopathol. (2012) 148:149–56. 10.1016/j.vetimm.2011.05.011 21620488

[81] Bartley K Huntley JF Wright HW Nath M Nisbet AJ. Assessment of cathepsin D and L-like proteinases of poultry red mite, *Dermanyssus gallinae* (De Geer), as potential vaccine antigens. Parasitol. (2012) 139:755–65. 10.1017/S0031182011002356 22310226

[82] Hentze MW Muckenthaler MU Andrews NC. Balancing acts: molecular control of mammalian iron metabolism. Cell. (2004) 117:285–97. 10.1016/s0092-8674(04)00343-5 15109490

[83] Zhou G Kohlhepp P Geiser D Frasquillo Mdel C Vazquez-Moreno L Winzerling JJ. Fate of blood meal iron in mosquitoes. J Insect Physiol. (2007) 53:1169–78. 10.1016/j.jinsphys.2007.06.009 17689557 PMC2329577

[84] Hajdusek O Sojka D Kopacek P Buresova V Franta Z Sauman I. Knockdown of proteins involved in iron metabolism limits tick reproduction and development. Proc Natl Acad Sci USA. (2009) 106:1033–8. 10.1073/pnas.0807961106 19171899 PMC2633537

[85] Kopácek P Zdychová J Yoshiga T Weise C Rudenko N Law JH. Molecular cloning, expression and isolation of ferritins from two tick species-*Ornithodoros moubata* and *Ixodes ricinus*. Insect Biochem Mol Biol. (2003) 33:103–113. 10.1016/s0965-1748(02)00181-9 12459205

[86] Vaughan JA Sonenshine DE Azad AF. Kinetics of ingested host immunoglobulin G in hemolymph and whole body homogenates during nymphal development of *Dermacentor variabilis* and *Ixodes scapularis* ticks (Acari: Ixodidae). Exp Appl Acarol. (2002) 27:329-40. 10.1023/a:1023347930746 12797408

[87] Hajdusek O Almazan C Loosova G Villar M Canales M Grubhoffer L. Characterization of ferritin 2 for the control of tick infestations. Vaccine. (2010) 28:2993–8. 10.1016/j.vaccine.2010.02.008 20171306

[88] Galay RL Miyata T Umemiya-Shirafuji R Maeda H Kusakisako K Tsuji N. Evaluation and comparison of the potential of two ferritins as anti-tick vaccines against *Haemaphysalis longicornis*. Parasit Vectors. (2014) 7:482. 10.1186/s13071-014-0482-x 25306467 PMC4197249

[89] Parizi LF Utiumi KU Imamura S Onuma M Ohashi K Masuda A. Cross immunity with *Haemaphysalis longicornis* glutathione S-transferase reduces an experimental *Rhipicephalus* (*Boophilus*) *microplus* infestation. Exp Parasitol. (2011) 127:113–8. 10.1016/j.exppara.2010.07.001 20619263

[90] Valle MR Guerrero FD. Anti-tick vaccines in the omics era. Front Biosci. (2018) 10:122–36. 10.2741/e812 28930608

[91] Lambertz C Chongkasikit N Jittapalapong S Gauly M. Immune response of *Bos indicus* cattle against the anti-tick antigen Bm91 derived from local *Rhipicephalus* (*Boophilus*) *microplus* ticks and its effect on tick reproduction under natural infestation. J Parasitol Res. (2012) 2012:907607. 10.1155/2012/907607 23213489 PMC3507137

[92] Hope M Jiang X Gough J Cadogan L Josh P Jonsson N. Experimental vaccination of sheep and cattle against tick infestation using recombinant 5'-nucleotidase. Parasite Immunol. (2010) 32:135–42. 10.1111/j.1365-3024.2009.01168.x 20070827 PMC2821529

[93] McKenna RV Riding GA Jarmey JM Pearson RD Willadsen P. Vaccination of cattle against the *Boophilus microplus* using a mucin-like membrane glycoprotein. Parasite Immunol. (1998) 20:325–36. 10.1046/j.1365-3024.1998.00149.x 9717194

[94] Almazán C Moreno-Cantú O Moreno-Cid JA Galindo RC Canales M Villar M. Control of tick infestations in cattle vaccinated with bacterial membranes containing surface-exposed tick protective antigens. Vaccine. (2012) 30:265–72. 10.1016/j.vaccine.2011.10.102 22085549

[95] Rodríguez-Mallon A Encinosa PE Méndez-Pérez L Bello Y Rodriguez Fernandez R Garay H. High efficacy of a 20 amino acid peptide of the acidic ribosomal protein P0 against the cattle tick, *Rhipicephalus microplus*. Ticks Tick Borne Dis. (2015) 6:530–7. 10.1016/j.ttbdis.2015.04.007 25958782

[96] He Y Rappuoli R De Groot AS Chen RT. Emerging vaccine informatics. J Biomed Biotechnol. (2010) 2010:218590. 10.1155/2010/218590 21772787 PMC3134832

[97] de la Fuente J Almazán C Blouin EF Naranjo V Kocan KM. RNA interference screening in ticks for identification of protective antigens. Parasitol Res. (2005) 96:137–41. 10.1007/s00436-005-1351-5 15824899

[98] Guerrero FD Miller RJ Pérez de León AA. Cattle tick vaccines: many candidate antigens, but will a commercially viable product emerge? Int J Parasitol. (2012) 42:421-7. 10.1016/j.ijpara.2012.04.003 22549026

[99] de la Fuente J Blouin EF Manzano-Roman R Naranjo V Almazan C Perez de la Lastra JM. Differential expression of the tick protective antigen subolesin in *Anaplasma marginale-* and *A. phagocytophilum*-infected host cells. Ann N Y Acad Sci. (2008) 1149:27–35. 10.1196/annals.1428.056 19120168

[100] Tuckow AP Temeyer KB. Discovery, adaptation and transcriptional activity of two tick promoters: construction of a dual luciferase reporter system for optimization of RNA interference in *Rhipicephalus* (*Boophilus*) *microplus* cell lines. Insect Mol Biol. (2015) 24:454–66. 10.1111/imb.12172 25892533

[101] Buchthal J Evans SW Lunshof J Telford SR Esvelt KM. Mice against ticks: an experimental community-guided effort to prevent tick-borne disease by altering the shared environment. Philos Trans R Soc Lond B Biol Sci. (2019) 374:20180105. 10.1098/rstb.2018.0105 30905296 PMC6452264

[102] Suarez CE Bishop RP Alzan HF Poole WA Cooke BM. Advances in the application of genetic manipulation methods to apicomplexan parasites. Int J Parasitol. (2017) 47:701–10. 10.1016/j.ijpara.2017.08.002 28893636

[103] de la Fuente J Antunes S Bonnet S Cabezas-Cruz A Domingos AG Estrada-Peña A. Tick-pathogen interactions and vector competence: identification of molecular drivers for tick-borne diseases. Front Cell Infect Microbiol. (2017) 7:114. 10.3389/fcimb.2017.00114 28439499 PMC5383669

