# Corrigendum: Understanding Tick Biology and Its Implications in Anti-tick and Transmission Blocking Vaccines Against Tick-Borne Pathogens

**DOI:** 10.3389/fvets.2020.00575

**Published:** 2020-08-31

**Authors:** Biswajit Bhowmick, Qian Han

**Affiliations:** ^1^Key Laboratory of Tropical Biological Resources of Ministry of Education, Hainan University, Haikou, China; ^2^Laboratory of Tropical Veterinary Medicine and Vector Biology, School of Life and Pharmaceutical Sciences, Hainan University, Haikou, China

**Keywords:** anti-tick vaccines, *Ixodes*, *Borrelia*, transmission-blocking, blood, saliva

In the original article, there was a mistake in the legend for Figure 1. The Figure was adapted from two sources, which the authors neglected to mention. The correct legend appears below.

**Figure 1:**
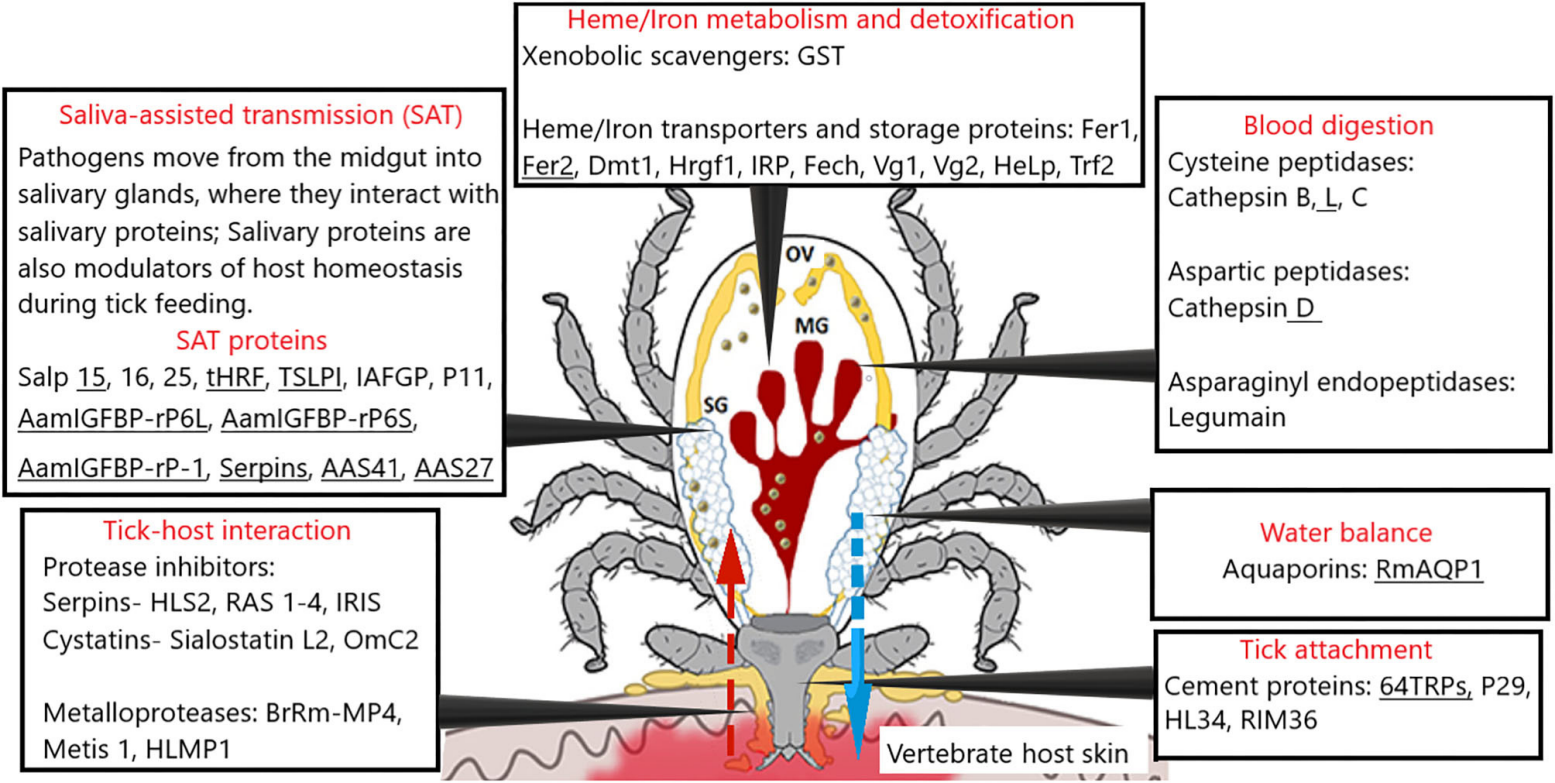
A schematic representation of tick physiological processes and involved molecules tested as vaccine candidates [modified from (78, 103)].

The authors apologize for this error and state that this does not change the scientific conclusions of the article in any way. The original article has been updated.
